# How Do We Recognize the Effects of Dairy Cattle Activity in the Lying Area? A Case Study in Free-Stall Barns

**DOI:** 10.3390/ani15060880

**Published:** 2025-03-19

**Authors:** Marek Gaworski

**Affiliations:** Department of Production Engineering, Institute of Mechanical Engineering, Warsaw University of Life Sciences, 02-787 Warsaw, Poland; marek_gaworski@sggw.edu.pl; Tel.: +48-22-593-45-83

**Keywords:** dairy cattle, free-stall barn, neck rail, partition, shine

## Abstract

Dairy cattle kept in barns with a free-stall system have contact with the structural elements of the stalls in the lying area. As a result of the cattle entering the lying stall, lying down, and standing up, shiny surfaces appear on the neck rails and partitions. These shiny surfaces can be a source of information about the activity of the animals in the lying stalls over a long period of time. This case study aimed to assess the diversity of shiny spots on the neck rails and partitions in the lying area of barns with a free-stall system. The length of the shiny surfaces and their locations on the neck rails and partitions were measured in three barns (on one dairy farm) containing a total of 512 lying stalls. The differences in the lengths of the shiny surfaces on the left and right partitions were assessed, and their locations on the partitions and neck rail were compared. Identifying the interactions between animals and metal elements of the lying stalls could contribute to improving the welfare of dairy cattle.

## Simple Summary

Due to long-term contact with dairy cattle, visual signs like a shiny surface can be recognized on the neck rails and partitions of lying stalls in free-stall barns. Shiny surfaces on the structural elements of the stalls and changes in the parameters of these stalls can provide important information on the behavior of dairy cattle, their lying conditions, and the maintenance of the stalls in the barn. This study aimed to evaluate the stalls in terms of the changes in their dimensions, as well as shiny surfaces on the neck rails and partitions, to assess the effect of the stall design on the interaction between dairy cattle and the stall.

## 1. Introduction

The lying area in a barn with a free-stall system affects the welfare of dairy cattle and can be the focus of research assessing their housing conditions. Many criteria can be used to assess the lying area in free-stall barns such as those related to the animals and the equipment in the area designated for rest.

The behavior of the dairy cattle, such as their lying and standing times, the number of bouts, and their preference for some lying stalls, are among the most important factors in recognizing the conditions for animals in the lying space [1]. Knowledge of the space requirements for dairy cattle comes from observations in which the standing up and down movements were studied [2]. The kinematic aspects of standing movements indicate the space needed by dairy cattle in the lying stalls [3], which facilitate the animals when performing their natural behaviors in the resting area [4].

The space available to a cow in a lying stall is a result of its construction and size. The size of the lying area may determine the dairy cattle’s behavior, especially the lying time. The smaller lying area of a tie stall, compared to the larger lying area in pens, may translate into shorter lying times [5,6]. The stall’s width affects the cows’ behavior when in contact with the stall surface and partitions. Still, it is unclear whether the animals judge or remember this aspect of the lying space when deciding where to lie [7]. Experiments have shown that cows spend more time lying down and have more prolonged bouts in wider stalls. It has been suggested that this could be explained by the fact that cows have less chance of contacting the partitions in these stalls. The contact with the partitions may only affect the duration of lying bouts in stalls with smaller widths [7]. Thus, the studies related to the behavior and preferences of cows in the lying area of barns with a free-stall housing system focused on various aspects of the contact of animals with the equipment of lying stalls [8].

Assessing the contact between cows and the equipment of the lying stalls can be used to answer specific research questions, such as the following: What is the frequency of contact between dairy cows and the restrictive elements in their housing environment [9]? Can contact with the partitions in the lying stall reduce the quality of rest and hinder the movement of cows [10]? Can the configuration of lying stalls, such as a tie-stall system, which obstructs free movement, affect the risk of injuries to dairy cows [11]? Can the installation of additional equipment in the space of the lying stalls mitigate the contact of cows with the metal parts of the stalls [12]? In barns with a free-stall housing system, how do animals interact with a free-stall infrastructure without constraints (i.e., tethers) [7]?

A cow entering a lying area and taking a lying position should remain in harmony with the given environment, which is one of the conditions for its well-being [13]. This harmony in the lying area may include contact with elements of the equipment, i.e., the neck rail and side partitions. A visible sign of this contact is a shiny area on the neck rails and partitions [14], which can be interpreted based on the dimensions of the lying stalls [8]. Shiny areas on the neck rails and partitions can provide valuable information about the animal’s activity in lying areas. This raises a research problem of assessing the shine parameters on neck rails and partitions, which can be used for further analysis.

This study assessed the variations in shiny surfaces on neck rails and partitions in the lying area of free-stall barns. The results of this assessment could provide a basis for further discussions on the contacts between dairy cattle and the equipment in lying stalls and the interpretation of animal activity in the lying area.

The following research hypothesis was formulated: The measured shiny surface parameters (length and location) differ significantly between the left and right partitions of the lying stall and on the neck rail. Demonstrating potential differences in shine parameters could be used to infer trends in the behavior of animals in the lying stalls, especially if these stalls differ in width and length, which may affect the parameters of the assessed shiny surfaces. Differences between shine parameters on the left and right partitions and the neck rail may result from the animals positioning themselves in the lying stall perpendicularly and at an angle to the rear curb. The animals may position themselves at an angle due to the design of the lying stall. This design can be improved, including by altering the structural elements of the lying stall that the animals are in contact with, as indicated by the analyzed shiny surfaces, to improve animal welfare [8]. The importance of the interactions between dairy cattle and the elements of the lying stall structure is expressed by the force of the animal’s impact, pressure, and rubbing on the neck rail and partitions. This impact force could reflect the discomfort of the dairy cattle, which could be used as an indication of their well-being. Both young animals [15] and cows [16] hit the neck rail and partitions, so the results of these types of analyses could be applied to many groups of animals.

Studies on dairy cattle welfare use cameras [12], data loggers [17], and other monitoring and vision systems that require special maintenance are vital. Shiny surfaces on the neck rails and partitions of lying stalls can be used to assess the contact of animals with these surfaces over a long period and are visually recognizable, facilitating their measurement. These non-invasive measurements can be used to assess animal behavior and preferences and, in the next future, to identify animal welfare factors and management practices to improve their welfare.

Measurements of shiny surfaces on neck rails and partitions may fill the gap in the development of non-invasive methods for assessing animal interactions with technical infrastructure in areas with lying stalls. Studies involving shiny surfaces may fill a gap in evaluating cattle behavioral effects in lying stalls as an extension of studies focusing solely on animal behavior.

Studies of the shiny surfaces on neck rails and partitions can be used to improve the design of lying spaces for dairy cattle and their welfare since they can provide practical knowledge about animal behavior in lying spaces. This knowledge can also be used to identify potential sources of discomfort for dairy cattle in free-stall housing systems.

## 2. Materials and Methods

**Figure 1:**
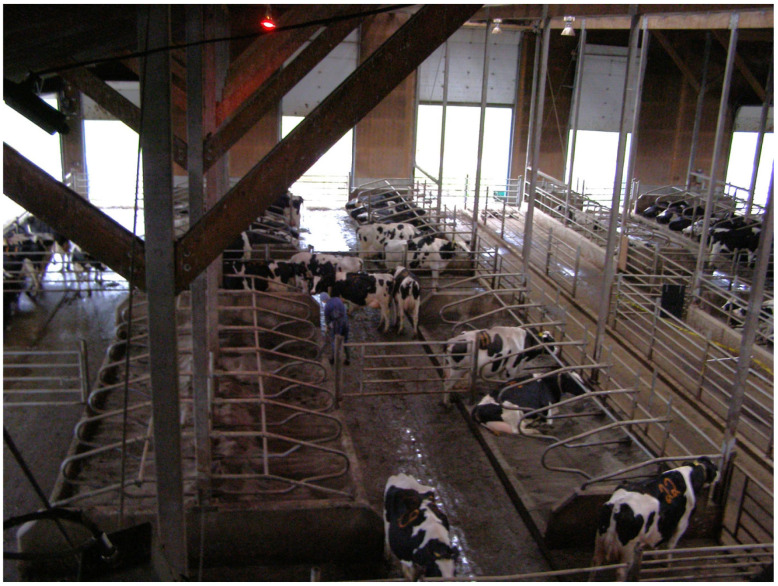
Part of the main barn with the layout of the pens and lying stalls in the pens.

The visible shine on the surface of the neck rails and partitions in lying stalls was assessed in three facilities with a free-stall housing system for various groups of dairy cattle. These facilities (two barns and a heifer barn) were on the same dairy farm: the University of British Columbia’s Dairy Education and Research Centre in Agassiz (Vancouver, BC, Canada). The cattle facilities differed in many details, including the year when the barns were built, the animals housed in them, the number of lying stalls, and their layout in the buildings. The oldest main barn (housing cows since July 2000) consisted of 288 stalls. The area with lying stalls was divided into smaller units (pens) of 12, 24, or 36 stalls, depending on the number of animals in one technological group. Each modular pen with twelve lying stalls consisted of three rows of four stalls. The two rows facing each other were open at the front (“head-to-head”) and had a total bed length of 240 cm per stall. The third row of lying stalls was adjacent to the concrete wall, and the stall bed was 30 cm longer than in the head-to-head rows. In each row, one of the outermost lying stalls adjoined the fence (with a tubular structure), and the other outer stall adjoined the concrete wall. The height of the neck rail and partitions above the bedding material level varied during the research period due to sand losses. The individual lying stalls were separated by Artex Y2K partitions (Artex Fabricators Inc., Langley, BC, Canada). The bedding material in all the lying stalls was river sand, sieved through a 2 mm sieve, washed with water to remove any silt, and stored outside until use. In the five modular pens (with twelve lying stalls per modular pen) in the oldest barn, younger cattle (pregnant heifers) were kept. In the same barn, 19 modular pens housed dairy cows. Figure 1 shows part of the main barn, showing the layout of the pens and lying stalls in the pens.

**Figure 2:**
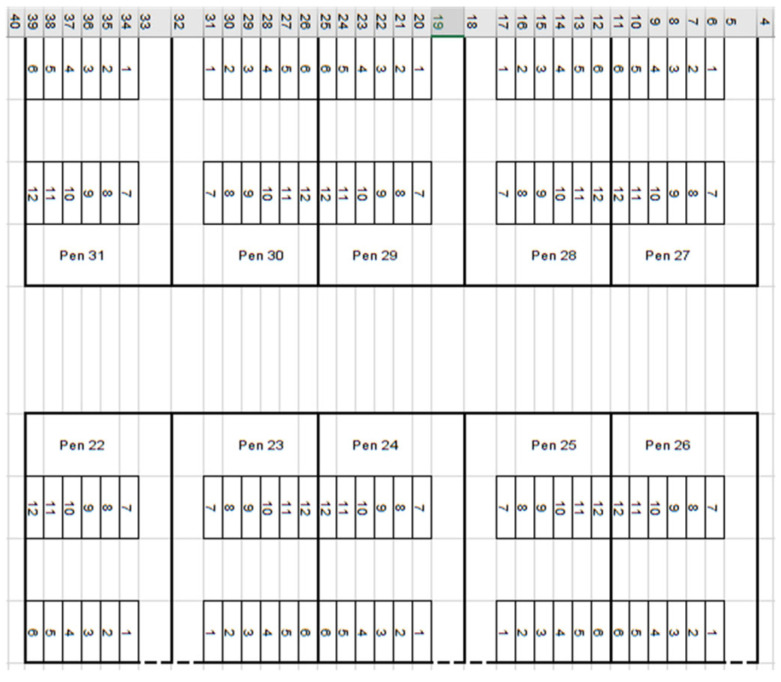
The locations and layouts of the pens and lying stalls in the pens in the Insentec barn; the diagram was created in Excel.

The second barn, called Insentec, opened in February 2005 and consisted of 120 lying stalls divided into 10 pens, with 12 lying stalls per pen. Each pen had two independent rows of six lying stalls. The stalls in each row were open at the front (no wall). One of the end stalls in each row was adjacent to a fence constructed of horizontal pipes. In the row closer to the feed alley, the animals stood with their heads toward the feed alley. In the second row, the cattle had their heads facing the opposite direction. Similarly to the oldest barn, the lying stalls were covered with sand. Two groups of dairy cattle were kept in the barn, i.e., dairy cows (used for milk production) and dry cows. The location of the 10 pens and the layout of the lying stalls in the individual pens in the Insentec barn are shown in Figure 2; the lying stalls in the rows are marked with numbers 1–6 and 7–12.

**Figure 3:**
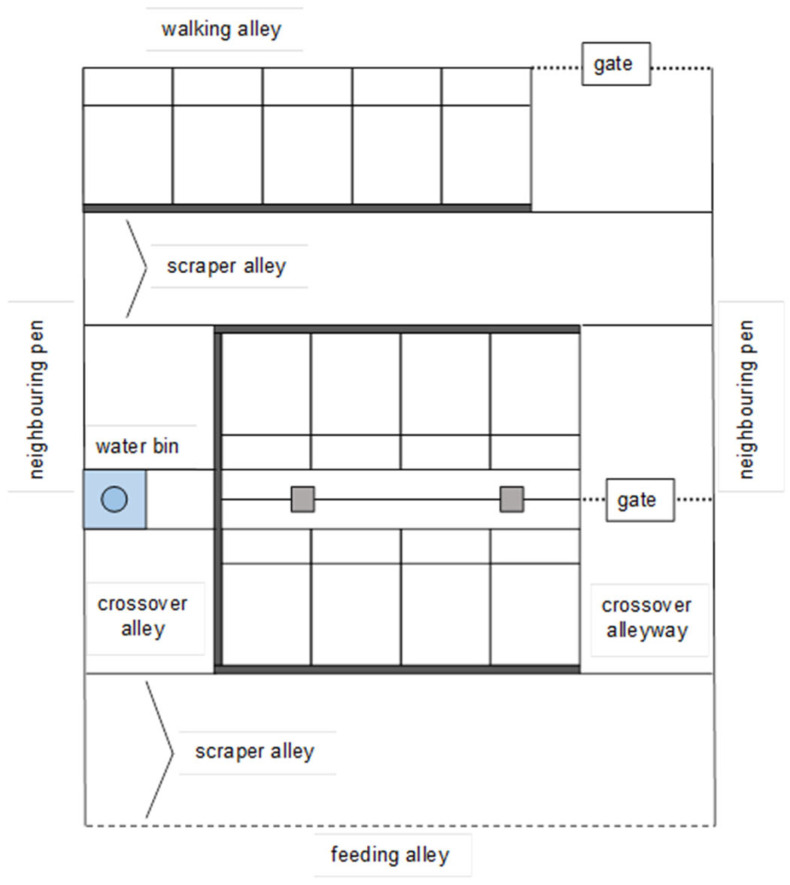
Diagram of a pen with 13 lying stalls in the heifer barn.

The third livestock facility included in the research was a barn for heifers, which was built in 2006. The part of the barn for heifers used in this study contained 8 pens, with 13 lying stalls per pen, totaling 104 stalls. The building had more pens, but the youngest animals were kept in an area without individual lying stalls. The lying area with the evaluated stalls for heifers was covered with sand. A diagram of a pen with 13 lying stalls in the heifer barn is shown in Figure 3.

The following acronyms were used to facilitate the measurement data management in the study: B.1—main barn; B.2—Insentec barn; and B.3—heifer barn.

The cows and heifers in the barns had free access to drinking water. The main barn (B.1) had group drinking bowls, one for every two pens. In barn B.2, the animals used individual drinkers, which were used to measure the amount of water intake. In barn B.3, there was one collective drinking bowl per pen. The drinking bowls were maintained in such a way as to ensure access to high-quality water. The groups of mature animals were fed a total mixed ration (TMR), which was available ad libitum. The TMR feed was delivered twice daily, starting from around 05:30 and 15:30. Cows were milked in side-by-side milking parlors in a 2 × 12 configuration. Morning and afternoon milking started at 5:00 and 15:00, respectively. Including the time spent milking, the cows were out of their pens for 60 to 120 min a day.

The cows and heifers were kept in barns throughout the year. Only for experimental purposes were some cows and pregnant heifers kept on a pasture during the spring and summer. The dairy farm kept enough animals to make the best possible use of the lying stalls in the barns for cows and heifers. The farm preferred not to keep the herd overstocked due to the number of available stalls.

The health status of the animals on the farm was systematically monitored. If necessary, a veterinarian intervened. Adult animals underwent hoof trimming twice a year. Mastitis, as well as diseases of the digestive system, were treated following the required procedures. Special care was provided to cows with lameness.

The aim was to measure all the stalls in the barns for cows and heifers in the dairy farm under study, i.e., the 512 lying stalls. The visible shiny surfaces on the neck rail and partition pipes in each stall were measured. These surfaces resulted from the animals coming into contact with these parts when they visit and occupy the lying stalls over a long period of time.

**Figure 4:**
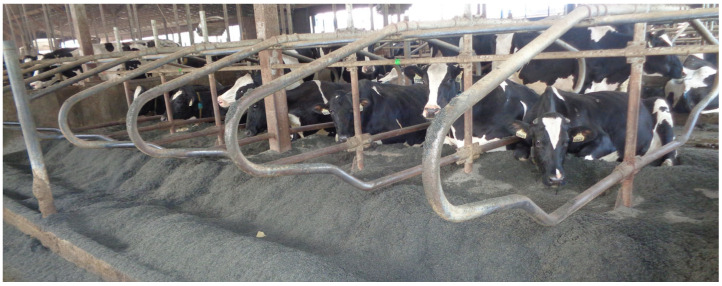
Neck rails with shiny surfaces in a row of lying stalls in barn B.1.

In the lower part of the neck rail, three zones could be observed, i.e., the left dirty part (matted), the middle part with a visible shiny area, and the right dirty part (matted). These three parts were used to determine the shiny surface parameters (shiny surface length and location) based on indirect measurements. The shiny surface length was calculated as the difference between the width of the neck rail and the width of the left and right matted parts. The distances of the shiny surface to the left and right ends of the neck rail in each lying stall were also determined. The width of the neck rail in each lying stall was measured as the distance between the right and left tubes of the partitions connected to the neck rail. The view of the neck rails with shiny surfaces in the row of lying stalls in one of the barns where the measurements were taken is shown in Figure 4.

**Figure 5:**
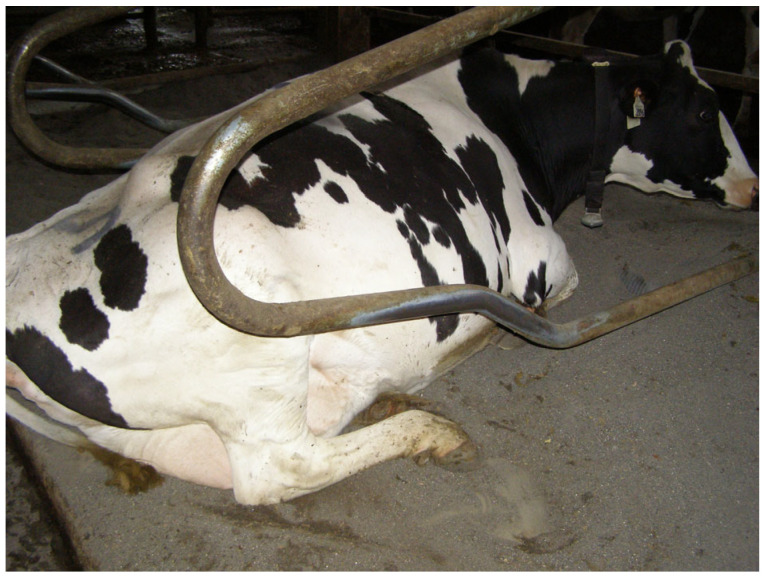
Visible shine on a partition between stalls in one of the barns resulting from the cow rubbing against the partition.

The measurements of visible shiny surfaces on the side partitions included two values. The first one was the length of the surfaces on both the left and right partitions (Figure 5). The length of the surface was measured in a straight line between the ends of the section. The shape of the partitions in the shiny area was irregular; therefore, to simplify and facilitate the measurements, the shortest distance (in a straight line) between the end edges of the shiny area was measured. In addition to the shiny surface length, the distance between the front edge of the shiny area and a reference point was measured. The reference point was where the lower part of the partition intersected with the vertical pillar supporting the metal structure of the stall. In each lying stall, measurements were carried out for the partitions on the left and right sides of the stall. Taking into account some differences in the construction of the lying stalls in the livestock facilities, the location of the reference point was different for each barn. As a result, it was impossible to directly compare the distances between the front edge of the shiny areas on the partition and the reference points in the different barns.

A flexible measuring tape with a stiffening function to facilitate taking measurements was used to measure all the parameters in the lying area. The accuracy of the measurements was 0.5 cm. The results of measurements in the lying area for cattle were entered into a previously prepared paper form. Data from the paper version of the form were then transferred to an Excel spreadsheet. The same person made all measurements at the lying stalls; to ensure a reliable measurement approach, some measurements were first taken together with another person to discuss how to delineate the shiny area on the neck rail and partitions. The measurements were made in the lying area while the cows were in the parlor so as to not disturb their daily routine. In the pens with dried cows and heifers, the measurements in the lying stalls were carried out during feeding so that as many animals as possible were outside the lying area. The intention of this approach was to minimize disturbances to the rest of the animals in the lying stalls.

The measurements on the neck rails and partitions of the lying stalls in the three barns were carried out in November and December when the temperature was low and the air humidity was high.

The data were analyzed using Statistica v.13 software (StatSoft Polska, Cracow, Poland). The descriptive statistical indicators, i.e., mean, standard deviation, minimum, and maximum values, were determined for the measured data: the stall width and the shiny surface length and location on the neck rail and partitions. The analysis, including comparing some data measured in the barns, was performed using the ANOVA test. The ANOVA test was used to assess the significance of the differences in the location of the shiny surfaces on the neck rail to the right and left pipes, which limit the width of the lying stall, and the differences in the width and location of the shiny surfaces on the left and right partitions of the lying stalls. The data for statistical analysis were prepared separately for each barn. The measurement data were included in the analysis as dependent variables. The independent variable was the side inside the lying stall where the shiny surface parameters were measured: the left side for the measurements on the left partition and the right side for the measurements on the right partition. The left and right sides also refer to the corresponding places on the neck rail. The assumed significance level was α = 0.05.

The measurement results were also used to show the changes in the length of the shiny part on the neck rail with respect to the width of the lying stall.

In summary, the measurement scheme included the following stages:

- Developing a preliminary measurement method based on observations conducted in pens with groups of dairy cattle;
- Identifying the parameters to be measured in the lying stalls;
- Schematic marking (numbering) of the lying stalls in individual pens in each barn;
- Developing a paper form for recording measurement data in the lying stalls;
- Consulting with another person on the correctness of the measurement method;
- Taking measurements using a measuring tape and recording the data on the paper form;
- Transferring the data from the paper form to a table in Excel;
- Preparing the data for statistical analysis and performing the analysis.

## 3. Results

**Figure 6:**
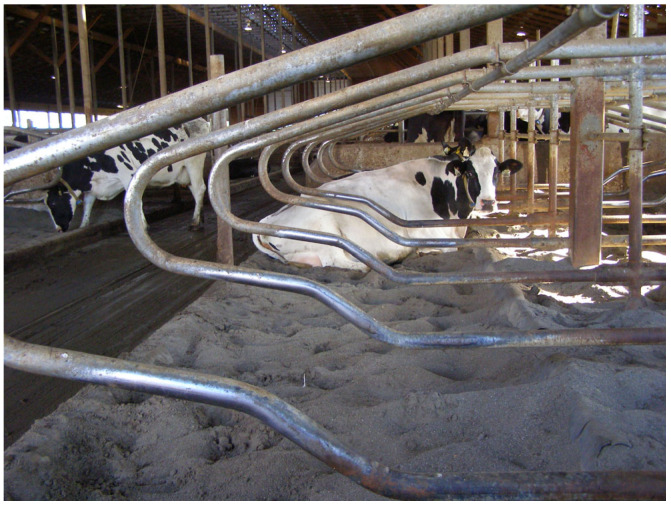
One of the rows of lying stalls ends with a concrete wall in barn B.1; the concrete wall prevented the identification of shiny surfaces.

The direct measurements of all the stalls in the three barns showed some differences in the number of useable measurements in each barn (Table 1). Usable measurements were those where a shiny surface could be identified and measured on the neck rail and partitions. In barn B.1, the lack of measurement results was because a shiny surface could not be identified on some partitions. In the B.3 heifer barn, shiny surfaces were also not identified on some partitions. However, there was another problem in facility B.3: one of the lying stalls was missing a neck rail. In barn B.1, a maximum of 2304 data points (288 lying stalls × 8 parameters) were collected. Considering some non-measurable parameters (158 data points) in barn B.1, it was possible to calculate the percentage of measured shiny surfaces, which was 93.14% (Table 1). The leading reason for being unable to measure a shiny area was the presence of a concrete wall (instead of a classic pipe partition) at the end of some rows with lying stalls (Figure 6). In addition, some of the end partitions were closed with a pipe fence instead of a typical pipe partition, and, in some cases, a shiny area was impossible to recognize. The only reason it was impossible to measure shiny surfaces on the partitions in the B.3 heifer barn was because of the concrete wall at the end of some rows with lying stalls; in this barn, there were 32 walls at the ends with stalls for younger heifers. The concrete walls in barn B.1 and heifer barn B.3 were painted white. As a result of contact with the bodies of the cows/heifers, it was possible to see some peeling paint (or dirty spots) on the concrete walls, but such effects were difficult to measure and compare with the shiny area on the pipe partitions. Problems with measuring the shiny area on the neck rails and partitions of the lying stalls only occurred in barn B.1 and heifer barn B.3. In barn B.2, however, there were no problems with identifying and measuring shiny surfaces. There were two explanations for this situation: barn B.2 was relatively new (compared to barn B.1) and had no concrete walls at the ends of the rows.

Considering the set of parameters in Table 1, the width of the lying stalls in two places was analyzed. The width of the stalls was measured at the neck rail and between the partitions (in the part closest to the rear curb). The mean values (±SD) of the width of the stall at the neck rail for each barn were 114.2 ± 5.6, 115.5 ± 3.4, and 87.8 ± 5.5 cm in B.1, B.2, and B.3, respectively. The mean values (±SD) of the width between stall partitions were 113.6 ± 5.7, 116.7 ± 4.4, and 87.8 ± 6.0 cm in barns B.1, B.2, and B.3, respectively. The analysis of variance did not show any significant difference in the width of the lying stalls measured at the neck rail or between the partitions in the part closest to the rear curb. For the measurements in barn B.1, the *p*-value was 0.0830. In barn B.2, the analysis of variance showed a *p*-value of 0.1044; in barn B.3, it was even higher (*p*-value of 0.8571).

Table 2 gives the minimum and maximum values for each parameter and barn. For each barn, it was possible to compare the measured minimum and maximum values based on a single pen, a single stall, the exact location of the pen, a single row, or a set of rows and its exact location in the pens. In this way, it was possible to identify specific places in the barn with the largest or smallest differences in stall width and other parameters.

The width of the shiny area on the neck rail was (mean ± SD) 76.3 ± 10.6, 82.2 ± 8.6, and 61.9 ± 11.2 cm in barns B.1, B.2, and B.3, respectively. The width of the left matted parts on the neck rail was (mean ± SD) 19.4 ± 7.1, 15.8 ± 5.4, and 14.0 ± 6.0 cm in barns B.1, B.2, and B.3. The width of the right matted parts on the neck rail was (mean ± SD) 18.5 ± 6.4, 17.9 ± 5.5, and 12.0 ± 4.4 cm in barns B.1, B.2, and B.3. In the analysis of variance of the lengths of the matted parts on the left and right sides of the neck rail in barn B.1, no significant difference was found (*p*-value equal to 0.0591). However, a significant difference was found in barns B.2 and B.3, where the *p*-values were 0.0013 and 0.0075, respectively.

The pens in barn B.1 mostly included cow pens and five pens for older (pregnant) heifers. The shiny areas on the neck rails in the cow pens had a length (mean ± SD) of 75.4 ± 10.8 cm; in the heifer pens, this was 79.4 ± 9.5 cm.

**Figure 7:**
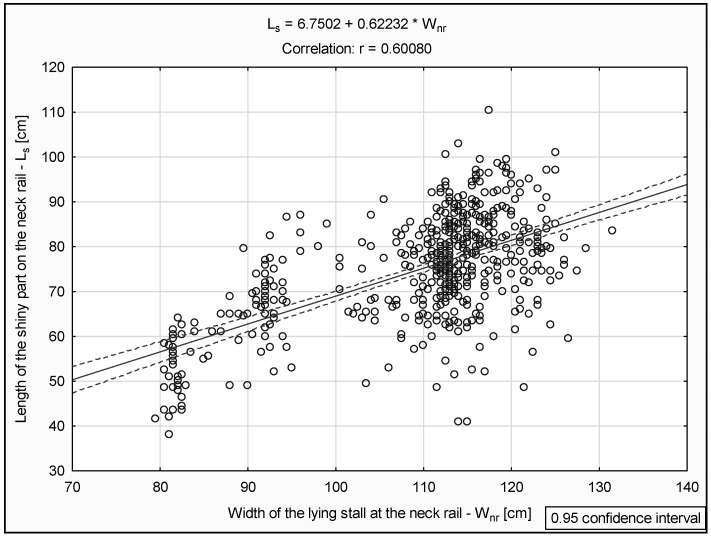
Relationship between the length of the shiny part on the neck rail (L_s_) and the width of the lying stall at the neck rail (W_nr_).

Figure 7 presents the length of the shiny part on the neck rail (L_s_) in relation to the width of the lying stall at the neck rail (W_nr_).

Considering the differences in the construction of the lying stalls in the barns, the measurement results for the location of shiny surfaces on the partitions could only be considered within individual barns. In barn B.1, the front location of the shiny area on the partitions on the left and right side was (mean ± SD) 91.8 ± 14.0 and 90.2 ± 15.0 cm, respectively. These parameters for barn B.2 were (mean ± SD) 119.7 ± 8.3 and 120.0 ± 7.2 cm. In the B.3 heifer barn, the shiny area on the left and right partitions (mean ± SD) was 72.0 ± 14.1 and 72.1 ± 14.1 cm from the front, respectively. The analysis of variance did not show any significant differences in the position of the shiny surface on the left and right partitions in the individual lying stalls. In every barn, the *p*-value was higher than 0.05.

The data from barn B.1 include results for 19 milking cow pens and 5 heifer pens. The front position of the shine on the left- and right-side partitions in the milking cow pens was (mean ± SD) 92.2 ± 14.0 and 90.3 ± 15.1 cm, respectively, while in the heifer pens, it was (mean ± SD) 90.3 ± 14.0 and 89.9 ± 15.0 cm, respectively. For the same measurements in barn B.1, it was possible to present data on the length of the shiny area on the left- and right-side partitions. In the case of pens with milking cows, the length of the shiny surface on the left- and right-side partitions was (mean ± SD) 68.3 ± 14.6 and 68.6 ± 14.4 cm, respectively, whereas in the pens with heifers, it was only (mean ± SD) 35.4 ± 10.5 and 35.7 ± 9.6 cm, respectively.

The length of the shiny surface on the left and right partitions in barn B.1 was (mean ± SD) 61.7 ± 19.2 and 62.2 ± 18.8 cm, respectively. In barn B.2, the lengths were (mean ± SD) 64.1 ± 9.0 and 62.9 ± 8.4 cm, respectively, and in heifer barn B.3, they were (mean ± SD) 55.6 ± 13.1 and 55.8 ± 13.9 cm, respectively. The analysis of variance did not find any significant differences in the length of the shiny surface on the left and right partitions in the individual lying stalls. For the measurement data from each of the three barns, the *p*-value was higher than 0.05.

## 4. Discussion

The presented approach to measuring the width of a stall, using three free-stall barns as an example, could lead to the use of “stall width” in research studies. The width of the stall measured at the neck rail and between the partitions may change during the period of use due to contact between the cows’ bodies and the partitions. The partitions may move during contact with the animals, changing their size, shape, attachment point (to other fixed elements), and tubular structure. Such movements could be recognized in flexible partitions in free-stall barns [18]. Still, it seems important to consider the possible movement of fixed partitions due to contact with cows. A high frequency of contact could translate into a possible change in the position of the partitions and, as a result, a change in the width of the stall between the partitions. Such considerations suggest including the stall width measured at the neck rail in the comparisons. However, in this area (the neck rail area), the cow only holds part of its neck, while most of the cow’s body is between the partitions, which indicate the animal’s comfort in the stall.

Considering the stall’s width, it is possible to calculate the individual space for the animal in the lying space, which can be used to assess the herd’s lying behavior and other behaviors of dairy cows kept in a free-stall barn [17]. Using this information, the following question can be asked: How can we recognize and assess the direct contact of an animal with the surrounding space, including the equipment? The individual space, limited to the lying space, offers an opportunity to analyze the effects of contact. A shiny surface on the neck rail and partitions can form due to contact and can be used to determine the different lying positions of the cattle in the stall based on their body contact with the partitions. The lying position could be subjected to detailed studies to better understand the probability of certain behaviors in the stall, e.g., with different types of partitions [15]. Studies have described stall designs without analyzing the lying position [19,20]. This aspect could be studied by assessing the visual shiny areas on the partitions.

Previous studies have focused on the contact of dairy cattle with the surrounding space, including the equipment, mainly from the animal’s point of view. Many detailed studies have identified the sources of health problems in dairy cattle, such as hock injuries [21,22], lameness [23,24], hoof health [25], neck injuries [26], and others. The health problems resulting from interactions with the surrounding space [27] can be identified by considering the importance of the environment, such as the type of bedding material and surface [28], the kind of floor [29], the design of the facility [30], and others. Further work on assessing the contacts and interactions of animals with the environment of the barn can be considered. These results could help in improving the design, regulations, and other technical factors for the effective use of the free-stall system and maintaining a high level of animal welfare.

The width of the lying stall is one of the considerations when designing free-stall systems [31], which is compared to the current housing conditions in dairy cattle production [32]. The recommendations regarding the width of the lying stall are based on the size of the animals: the width should be about twice the width of the hips, which translates into a width of about 100–120 cm [33]. The width of the lying stall measured at the neck rail in this study was (mean ± SD) 114.2 ± 5.6 cm in barn B.1 and 115.5 ± 3.4 cm in barn B.2. Both values are within the recommended range (100–120 cm). The difference in width between the two barns (B.1 and B.2) was 1.3 cm. However, when we compared the stall width between the partitions, the difference (for barns B.1 and B.2) was 3.1 cm.

A wider space between stall partitions provides more space for dairy cattle, making it more comfortable for the animals to stand throughout the stall without touching the partitions or neck rail [7]. Based on the observations from this study, further research could determine whether wider stalls are associated with the formation of shiny areas or lack thereof on stalls. Such research could determine the mechanism of shine formation on stalls (e.g., whether it is from when cows enter the stall, stand, lie down, or stand up).

The cow should be able to stand up without hitting the neck rail, and a polished (shiny) underside may indicate that it is incorrectly positioned [34]. In this way, the neck rail’s shiny underside provides an important source of information about the cows’ behavior in the free-stall area. The measurements of the shiny part on the neck rail showed that the percentage of this part in the width of the stall was (mean ± SD) 66.8 ± 9.2%, 71.0 ± 7.2%, and 70.1 ± 9.7% in barns B.1, B.2, and B.3, respectively. It was also possible to compare the actual width of the shiny area on the neck rail: in barns B.1 and B.2, it was (mean ± SD) 76.3 ± 10.6 and 82.2 ± 8.6 cm, respectively, and in heifer barn B.3, the width was smaller (61.9 ± 11.2 cm). When analyzing the data on the width of the shiny area on the neck rail, there were interesting results for some rows of lying stalls. For example, in barn B.2, the average width of the shiny area on the neck rail in two independent rows was exactly the same, i.e., 82.2 cm. However, in barn B.1, in rows located near the feed alley and in the rows furthest from the feed alley, the width was (mean ± SD) 71.7 ± 10.5 and 80.6 ± 8.5 cm, respectively. Such differences from different places in the pen confirm the differences in the use of the lying stalls that were found in other studies [35].

Partitions are a key element in the construction of stalls in a free-stall barn since they create individual resting spaces for the animals. The appropriate construction, shape, and position of the partitions, which limit the stall space, are important for ensuring comfortable lying conditions. However, body contact with the partitions can reduce the animal’s comfort. In one detailed study [3], the maximum instantaneous velocity of body markers reached 220 cm/s, which indicated that the cows touched incorrectly placed stall partitions and lying surfaces with considerable force. In an experiment with pressure sensors placed on different stall parts, Blom et al. [16] found that the cows touched stall partitions more than 100 times daily. Considering the considerable body mass of the cow in contact with the partitions, the horizontal movement of the partition could result in a change in the stall width. However, such a hypothesis requires further research. The design and placement of stalls and other potential obstructions should consider displacement measurements to minimize disturbances to cow movements and prevent injury [3]. Some studies have shown shorter lying periods in response to potential discomfort during lying. Tucker et al. [7] reported shorter lying periods and daily lying times in cows housed in narrower stalls and suggested that contact with stall partitions may reduce lying comfort.

Measurements of shiny surfaces on neck rails and partitions allow for the assessment of animal activity over a long period of time. Shiny areas, which indicate areas with abrasive wear of the metal pipes, are generated by dairy cattle every day due to contact with the structure of the stall. It is unclear whether this contact occurs when the animals lie down or during standing and lying movements [7]. Regardless of the mechanism of shine formation on neck rails and partitions, the proposed approach for analyzing shiny surfaces as indicators of long-term animal behaviors provides an alternative solution to many trials, where animal behavior is studied over a short period of time (e.g., several weeks [35,36], one year [37], or several seasons [38]). The presented approach could be used for monitoring the behavior of dairy cattle in the lying area [39,40,41,42]. Another feature of the long-term use of equipment in the barn is the risk of gradual wear and tear potentially becoming damage [43]. Our study, conducted in three barns, identified damage to the structural elements of the stalls in the lying area, which could have been due to the contact of the dairy cattle with the metal equipment in the stalls [44].

The design of the lying stall, especially its steel pipes, can be a source of discomfort for animals due to contact (impact) with the fixed elements of the stall structure [45,46]. The shiny areas on the neck rail and partitions indicate that this contact results from the space limitation in the lying area due to the structural elements (pipes). If the construction elements of the lying area limit the freedom of the animals, such observations could aid in the search for alternative solutions. The study by Abade et al. [47] proposed using wooden boards protruding (8 cm) above the stall surface to partition individual lying areas. It was assumed that cows would prefer these less restrictive places and spend more time lying down and standing there. Although stall partitions define the lying area for individual cows [48], new stall designs are being developed for dairy cattle, including those with minimal stall partitioning [49]. A proposal to provide cattle with access to more open space in the lying area was to divide wide lying areas with hanging wooden partitions [50]. These alternative solutions were compared with classically constructed lying stalls using the key evaluation criteria of cleanliness and the behavior and comfort of the animals. Such criteria cannot include shiny areas on the elements of the stall construction, which limits the use of this method in this type of research analysis.

Shiny surfaces on neck rails and partitions provide important information about the contact of animals (dairy cattle) with the metal elements in a lying stall. The width and position of shiny spots may indicate how the animals position themselves in relation to the structural elements of the lying stall when they are lying down and standing up. However, the force with which the animal hits the neck rail and partitions is also important. Forceful contact with the structural elements of the lying stall has been observed in all groups of dairy cattle, including calves [15]. Hitting the neck rail or partition may be a source of discomfort for animals, which could affect their well-being in the designed housing system [51]. A criterion for designing lying stalls is their dimensions based on the body size of the animals and the lying behavior of cows [52] and other groups of cattle [53]. The dimensions of lying stalls can be adjusted by changing the position of the neck rail, which translates into increased cleanliness of the udders [54] and lying stall [55]. The surface of the shiny part and its position on the neck rail can provide additional information on the effects of the neck rail adjustment on the cattle’s behavior.

The measurements of shiny surfaces on neck rails and partitions can be used in simulation models to design lying stalls and lying areas in barns on a wider scale. The cleanliness and shiny areas of the neck rails and partitions should confirm that the dimensions of the stalls in free-stall barns are correct; however, a prerequisite for establishing the correct size of stalls for all animals is that the animals in the herd are of a uniform size [14]. The proper design of a bedding area, taking into account the known interactions of the animals with the structural elements, is one method for improving the welfare of dairy cattle by alleviating stress [56].

The location of shiny surfaces on partitions can provide information on the extent to which the design of the lying stall allows the animals to move too far to the front of the stall, which may translate into a risk of increased surface contamination with feces. The technical equipment of the lying stalls must not threaten the animals and must help maintain balanced housing conditions in the barn.

Differences in the width and position of the shiny part on the neck rail can be used to interpret the animal’s behavior in lying stalls. If there is no shiny area on the neck rail, this would suggest that the animals avoid the stall or that the stall is long enough for the animals to not interact with the neck rail. Reducing contact with the neck rail can increase the comfort of the cow, especially if the cow does not hit the neck rail with their neck when standing up. The ideal situation would be for the cow to not hit the neck rail when standing up, and, after standing up, for them to in a place where they can defecate into the manure alley and not in the lying stall. If the lying stall is contaminated with feces and, at the same time, there is no shine on the neck rail, this may indicate that the neck rail is too far from the rear curb. This is practical information for the farmer, indicating that the neck rail needs to be corrected, i.e., moved towards the curb, which will reduce the contamination of the bedding material in the lying stall with feces.

Similar considerations to those in the case of the neck rail can be developed for the side partitions in the lying stalls. If there is no shiny surface on the partitions or it is short, this may suggest limited contact of the animals with the partitions, possibly due to the lying stall being too wide. In a lying stall that is too wide, the animals may tend to position themselves diagonally [15], which increases the risk of the contamination of the bedding material with feces. Shiny surfaces or their absence on the partitions may provide practical information about the need to adjust the width of the lying stalls.

The measurements of shiny surfaces on the neck rails and partitions can be used to determine the correct dimensions of the lying stalls for dairy cattle. Our results are consistent with earlier considerations for lying stalls, which also emphasizes the importance of assessing shiny surfaces on the neck rails and partitions to confirm the correct dimensions for the stalls [57,58].

In the research on animal resting areas, welfare is of particular importance. Welfare refers to animals living in harmony with nature, and the first helpful definition includes the mental well-being and health of the animal [13]. The following question was formulated [59]: Can the welfare of dairy cattle be harmonious with dairy production technologies and its technical equipment? Thus, it is important to assess this harmony correctly. In the lying area for dairy cattle in free-stall barns, shiny features on the neck rail and partitions can signal the animals’ harmonious use of the lying stalls.

The measurement of shiny surfaces on neck rails and partitions in the lying areas is a non-invasive research method that allows for the assessment of the animal housing conditions that affect their welfare. In practice, using non-invasive methods for studying pain and chronic stress using infrared thermography [60] to assess animal welfare is a standard practice in herd management. Another non-invasive method is tear and saliva collection [61]. However, many non-invasive methods for studying animal welfare require the use of specialist equipment, while measurements of shiny surfaces on metal elements in the lying area involve simple tools that are available on dairy farms.

Identifying shiny surfaces on neck rails and partitions may be a simple tool for assessing the behavior of dairy cattle in their stalls over a long period. This method, which can help producers improve the comfort and general welfare of their animals, could become a tool in the effective management of dairy herds. This is particularly true for optimizing the accommodations and housing, stall designs, and space allowances [62].

However, the proposed method may have limitations. Such measurements cannot be performed in new barns where animals have just been introduced since shiny surfaces on metal structural elements of lying stalls can only be identified after long-term use of the barn. In general, measurements of shiny surfaces on metal neck rails and partitions are inconvenient and time-consuming and require adjustment to the daily rhythm of the animals to avoid disturbing their rest in the lying stalls. When measuring shiny metal surfaces on neck rails and partitions, adequate lighting is also important to facilitate the identification of the measured parameters. The ideal conditions would be under natural light. This light can be supplemented by artificial light when there is a need to illuminate the space in the barn. It is important to maintain, if possible, similar lighting conditions during the measurements. Barns may differ in access to natural light, so artificial lighting is required when measuring shiny surfaces in some facilities. On some farms, the free-stall lying area is equipped with neck rails made of tape or elastic materials (e.g., rope) with a plastic cover, which can make it more difficult to identify shiny surfaces, limiting the use of this method.

## 5. Conclusions

Shiny surfaces on neck rails and stall partitions in free-stall barns can provide valuable information about the activity of dairy cattle over a long period of time. Identifying the interactions between animals and metal stall components could contribute to research on improving the welfare of dairy cattle.

The length and position of the shiny surfaces on the neck rails and partitions of the lying stalls can provide important information for the farmer, such as the proper design and dimensions of the lying stalls based on the average size of the animals.

## Figures and Tables

**Table 1:** Number of unmeasured parameters in the lying stalls in the three studied barns.

Description		Number of Unmeasured Parameters		
		Barn B.1	Barn B.2	Heifer Barn B.3
Total number of lying stalls		288	120	104
Measured parameter	Width of the lying stall (at the neck rail)	0	0	0
	The matted part (at the neck rail), L-h	0	0	1
	The matted part (at the neck rail), R-h	0	0	1
	Width between partitions	0	0	0
	Shine on partition, position, L-h	41	0	8
	Shine on partition, length, L-h	39	0	8
	Shine on partition, position, R-h	40	0	8
	Shine on partition, length, R-h	38	0	8
Percentage of usable measurements (%)		93.14	100	95.91

Notes: L-h—left-hand side; R-h—right-hand.

**Table 2:** Minimum and maximum values of measured parameters (cm).

Description		Barn B.1		Barn B.2		Heifer Barn B.3	
		min.	max.	min.	max.	min.	max.
Measured parameter	Width of the lying stall (at the neck rail)	100.5	131.5	107.0	126.0	79.5	99.0
	The matted part (at the neck rail), L-h	0.0	42.0	0.0	27.0	0.0	30.0
	The matted part (at the neck rail), R-h	4.0	42.0	0.0	30.0	0.0	23.0
	Width between partitions	94.0	135.0	107.0	131.0	78.5	105.0
	Shine on partition, position, L-h	52.0	112.0	92.0	133.0	40.0	100.0
	Shine on partition, length, L-h	12.0	106.0	47.0	96.0	30.0	89.0
	Shine on partition, position, R-h	37.0	121.0	77.0	130.0	42.0	101.0
	Shine on partition, length, R-h	15.0	104.0	49.0	108.0	20.0	91.0

Notes: L-h—left-hand side; R-h—right-hand side.

## Data Availability

Data are contained within the article.
